# Association between systemic immune inflammation index and adolescent obesity in a cross-sectional analysis

**DOI:** 10.1038/s41598-025-91125-6

**Published:** 2025-02-22

**Authors:** Yu-zhen Zhang, Run-wei Ma, Suwas Bhandari, Juan Xie, Xiao-yu Zhang, Chao Xie, Hong Duan, Juan Meng, Qiong-yu Wu, Kai Liu, Bo Feng, Li-ming Cheng

**Affiliations:** 1 Department of Anesthesiology, Kunming Children’s Hospital, Yunnan, China; 2 Department of Cardiac Surgery, Fuwai Yunnan Hospital, Chinese Academy of Medical Sciences/Affiliated Cardiovascular Hospital of Kunming Medical University, Yunnan, China; 3 Wenzhou Medical University, Wenzhou, China; 4 Department of Comprehensive Pediatrics, Kunming Children’s Hospital, Yunnan, China; 5 Department of Cardiac Surgery, The First People’s Hospital of Kunming, Yunnan, China; 6 Surgical Intensive Care Unit, Kunming Children’s Hospital, Yunnan, China; 7 Department of Anesthesiology, Kunming City Maternal and Child Health Hospital, Yunnan, China

**Keywords:** Systemic immune-inflammation index, Adolescent obesity, NHANES, Inflammation, Endocrine system and metabolic diseases, Chronic inflammation, Obesity

## Abstract

Obesity is a prevalent health issue among adolescents, characterized by chronic low-grade inflammation, which increases the risk of developing various chronic diseases in the future. The systemic immune-inflammation index (SII) serves as an indicator of inflammation and immune response. This study conducted a cross-sectional analysis using data from the National Health and Nutrition Examination Survey (NHANES) from 2007 to 2016, including 5,676 participants. A multivariate logistic regression model, Generalized Additive Models (GAM), and subgroup analysis were used to examine the relationship between obesity and SII. The multivariate logistic regression results revealed a significant positive correlation between log SII and adolescent obesity (1.254 [1.024–1.537]). Furthermore, the risk of obesity increased with higher quartiles of SII. Subgroup analysis and interaction tests showed that this positive association persisted across various factors, including female gender, race (Non-Hispanic White and Mexican American), non-hyperlipidemia, normal white blood cell count, and PIR < 1. Additionally, a U-shaped relationship between log SII and obesity was observed, with a turning point at 6.410. The findings suggest that an increase in the systemic immune-inflammation index is significantly associated with obesity in adolescents. However, further validation through large-scale prospective studies is needed.

## Introduction

Adolescent obesity is a pervasive problem with significant health risks^1,2^. Overweight or obese children and adolescents are at a significantly increased risk of chronic diseases such as type 2 diabetes^3^, cancer^4,5^, or cardiovascular disease^6^ in adulthood. Additionally, adolescent obesity is associated with mental health issues^7^, with some studies indicating that obesity may lead to symptoms of depression, anxiety, and low self-esteem in adolescents^8^. Preventing obesity during adolescence is crucial for reducing the likelihood of chronic diseases in adulthood.

Obesity is closely related to chronic low-grade inflammation^9^. The large amounts of macronutrients in adipose tissue produces inflammatory molecules, such as TNF-α and IL-6. This leads to the infiltration of macrophages into adipose tissue and reduces the synthesis of adiponectin, forming a pro-inflammatory environment and oxidative stress^10,11^. These inflammatory factors can trigger a systemic inflammatory response, leading to metabolic abnormalities and cardiovascular diseases^12,13^. Therefore, studying the role of inflammation in obesity is crucial for better understanding and effectively controlling the associated health risks.

Blood platelets, neutrophils, and lymphocytes play important roles in the immune response to chronic inflammation and obesity^14^. Platelets not only participate in blood clotting but also promote chronic low-grade inflammation by interacting with immune cells^15^. Neutrophils, which are important immune cells, amplify the inflammatory response by releasing cytokines and chemokines, and their levels are elevated as part of the immune system’s response to ongoing inflammation^16^. Lymphocytes, responsible for maintaining immune surveillance, typically exhibit functional abnormalities in chronic inflammation, leading to a decrease in immune tolerance and possibly contributing to the persistence of inflammation^16^.

The Systemic Immune-Inflammation Index (SII) quantifies the relationship between platelet count, neutrophil count, and lymphocyte count^17^. This test has been used to determine the prognosis of various cancers, such as gastric^18^, esophageal^19^, and colorectal^20^. In this regard, it is the best tool to comprehensively reflect the balance between inflammatory factors and immune responses in the body. Some studies found that SII is related to other inflammatory diseases, including acute pulmonary embolism^21^, heart failure^22^, rheumatoid arthritis^23,24^, and chronic kidney disease^25^. However, so far, it remains unclear whether there is a potential relationship between adolescent obesity and SII. While SII has been widely studied in various adult diseases, its role in adolescent obesity has not been explored, making this study novel in examining this relationship in a population that has not been well studied.

Considering that adolescent obesity is influenced by a variety of complex factors, we selected the following covariates: age, gender, race, poverty-to-income ratio (PIR), activity level, white blood cell count, hyperlipidemia, and sedentary time. Age and gender are fundamental physiological variables that may influence both the onset of obesity and its association with immune response. Race and PIR reflect socioeconomic status, which is known to be closely linked to obesity, health behaviors, and inflammation levels. Activity level and sedentary time directly affect the development of obesity and associated inflammatory responses^26^. The inclusion of WBC as a covariate both reflects the overall immune response and controls for immune interferences, and distinguishes the biological differences between WBC and the cells used in SII, more accurately capturing the relationship between SII and obesity. The close relationship between hyperlipidemia and obesity and inflammation makes it an important modifier that may influence the relationship between SII and obesity. By considering these covariates, we aim to minimize potential confounding effects and ensure a more accurate assessment of the independent relationship between SII and adolescent obesity.

This study uses the data of National Health and Nutrition Survey (NHANES) to explore the relationship between SII levels and adolescent obesity and aims to investigate how various covariates may influence this association. By conducting a detailed analysis of the association between SII and adolescent obesity, we hope to reveal the potential of SII as a new inflammatory marker for predicting and intervening in obesity. This approach offers new insights into the prevention and control of adolescent obesity.

## Research subjects and methods

### Data and sample source

The primary data of this study comes from the NHANES, funded by the Centers for Disease Control and Prevention^27^. Nationally representative data were obtained using complex multi-stage sampling techniques. The website is as follows: https://www.cdc.gov/nchs/nhanes/.

**Fig. 1:**
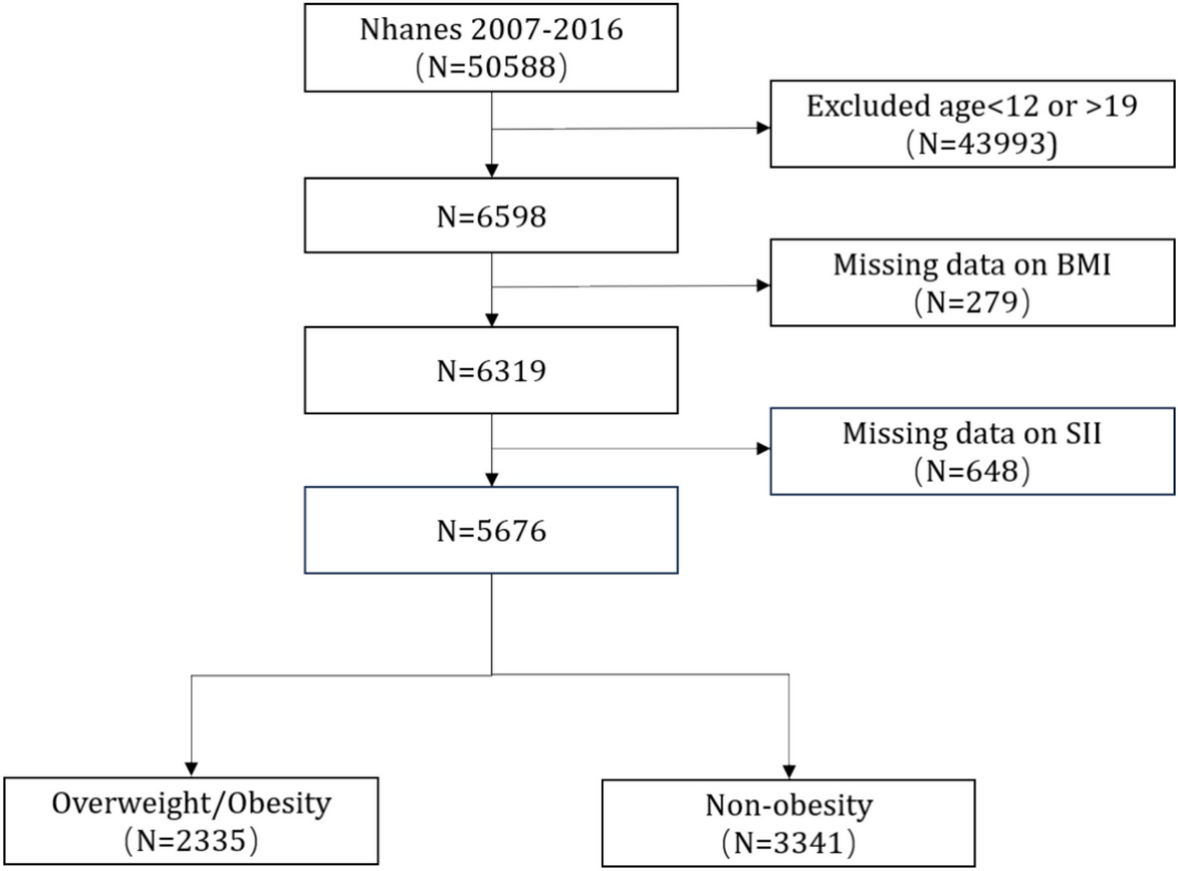
Flowchart of the participants selection from NHANES 2007–2016.SII, Systemic Immune-Inflammation index; BMI, body mass index.

Fifty thousand five hundred eighty-eight people participated in the five NHANES cycles from 2007 to 2016. We excluded 43,990 individuals under 12 or over 19 years old, 279 without BMI data, and 643 with missing SII data. All missing covariate data were predicted using the predictive mean matching method, and composite data were established based on other variable information. Finally, 5,676 subjects entered this study analysis. Specifically, 2,335 subjects with a body mass index (BMI) ≥ the gender/age-specific 85th percentile were classified into the obese group, which included both overweight and obese subjects. The remaining 3,341 subjects were classified into the non-obese group^28^. (Fig. 1).

### Exposure variables

SII = Platelet count × Neutrophil count / Lymphocyte count^29^. The distribution of the original value of SII in the study is skewed, showing a sizeable numerical range and extreme observations. To solve this skewness problem, we performed a logarithmic transformation on the SII value, forming a continuous variable: log SII. Logarithmic transformation also helps to compress the data range and reduce the impact of extreme values on statistical analysis, thereby improving the robustness of the results.

### Definition of other variables

PIR: An indicator to assess family economic status, calculated as total family income divided by the federal poverty line^30^. PIR value ≤ 1 indicates that the family is in poverty, and the higher the PIR value, the better the family's economic situation.

### Hyperlipidemia

Defined as a triglyceride level higher than 130 mg/dl in the blood according to the 2011 "Comprehensive Guidelines for Cardiovascular Health and Risk Reduction in Young Populations"^31^. The classification of hyperlipidemia is based on NHANES laboratory data.

### Physical activity

Defined as adolescents participating in vigorous or moderate-intensity physical activity for less than 10 min per week^32^.

### Variables

We carefully selected covariates that could affect the relationship between SII and obesity (24–26). The collected demographic data include age (years), gender (male/female), ratio of family income to poverty (PIR), race (Mexican American, Other Hispanic, Non-Hispanic White, Non-Hispanic Black and Other Race—Including Multi-Racial), sedentary time (minutes), white blood cell count (1000/mL), activity level (active/inactive), and hyperlipidemia (hyperlipidemia/non-hyperlipidemia) and other variables.

### Statistical analysis

In our study, considering the complex sampling design of NHANES and the different sampling weights, we used the appropriate weighting settings of NHANES to calculate the weighted average of continuous variables and their corresponding standard errors, and to determine the weighted percentage share of categorical variables. This approach was used to more accurately estimate the overall level and infer the error, ensuring the accuracy and representativeness of the analysis results. We compared the baseline characteristics of obese and non-obese groups. Then, we used weighted t-tests and chi-square tests to evaluate the differences between these two groups. To further assess the sensitivity of SII in both groups, we additionally established a multivariate linear regression model.

Next, to study the relationship between SII and obesity, we established three logistic regression models—Model 1, without adjusting for any covariates; Model 2, adjusted for age, gender, and race; Model 3, adjusted for age, gender, race, poverty-to-income ratio (PIR), activity level, white blood cell count, hyperlipidemia, and sedentary time. In the models, because the distribution of SII data did not follow a normal distribution, we performed a log-transformation of SII and used quartiles of SII for sensitivity and trend tests. We also analyzed the threshold effect and used a generalized additive model (GAM) to fit the smooth curve to evaluate the nonlinear relationship between log SII and obesity. In addition, through subgroup analysis and interaction tests, we studied the relationship between log SII and obesity in different subgroups. After the subgroup analysis, we applied the Benjamini-Hochberg (BH) method to adjust the p-values for controlling the false discovery rate (FDR).

### Threshold effect analysis

We fitted a generalized additive model (GAM) to plot a smooth curve and observed the nonlinear relationship between log SII and obesity, identifying the turning point. The turning point was determined by finding the value of log SII that maximized the model's logarithmic Odds values, which indicates the point where the relationship between log SII and obesity changes most significantly. Subsequently, we used a two-stage regression model to study the relationship between log SII and adolescent obesity before and after the turning point. The significance threshold was set at P < 0.05.

### Statistical significance

All statistical analyses are two-way; the significance threshold is P < 0.05. The results are expressed as odds ratios (OR) and their corresponding 95% confidence intervals (CI).

### Mediation analysis

We used the lavaan package in R to construct a path analysis model based on structural equation modeling (SEM) to evaluate the direct effects of the Systemic Inflammation Index (SII) on obesity, as well as the indirect effects through mediating variables. Path coefficients were estimated using the Maximum Likelihood (ML) method, and 1,000 bootstrap resamples were performed to generate standard errors and 95% confidence intervals for the indirect effects, ensuring the robustness of mediation effect testing. To improve model accuracy, all covariates were controlled in the analysis. By standardizing the path coefficients, we further quantified the contributions of each path to the total effect and decomposed the direct and indirect effects of SII on obesity. This approach provides a reliable quantitative framework for analyzing the complex relationships between SII, inflammatory markers, and obesity.

## Results

### Baseline characteristics of participants

A total sample of 5676 American adolescents aged between 12 and 19 participated; the typical mean age is 15.43 ± 0.04 years. Of the total, 52.03% were males and 47.97% were females. The average SII score for all the participants is 465.87 ± 5.58; WBC is 6.94 ± 0.05; PIR is 2.51 ± 0.06; sedentary time was 466.56 ± 4.59 min.

Based on the baseline analysis of the population, we found that obese American adolescents exhibited significantly higher SII scores, higher white blood cell counts, less physical activity, longer sedentary time, higher prevalence of hyperlipidemia, and lower PIR. There was a significant racial difference between the two groups (P < 0.001), with the obese group having a lower proportion of non-Hispanic whites and a higher proportion of Mexican Americans and non-Hispanic Blacks (Table 1). These racial and socioeconomic differences, such as the lower poverty-to-income ratio (PIR) among obese adolescents, may contribute to the observed disparities in obesity rates. The higher prevalence of obesity among Mexican Americans and non-Hispanic Blacks may reflect underlying social and economic factors, such as access to healthcare, dietary habits, and opportunities for physical activity.

**Table 1:** Basic characteristics of participants (n = 5,676) in the NHANES 2007–2016.

Variable	Total (n = 5676)	Obesity (n = 2335)	Non-obesity (n = 3441)	p Value
Age(years)	15.43 ± 0.04	15.33 ± 0.06	15.50 ± 0.06	p = 0.088
SII	465.87 ± 5.58	515.25 ± 9.61	433.79 ± 6.43	p < 0.001
Log SII	6.00 ± 0.01	6.12 ± 0.02	5.93 ± 0.01	p < 0.001
WBC	6.94 ± 0.05	7.51 ± 0.08	6.57 ± 0.06	p < 0.001
PIR	2.51 ± 0.06	2.29 ± 0.06	2.65 ± 0.07	p < 0.001
BMI	24.00 ± 0.13	29.62 ± 0.16	20.36 ± 0.06	p < 0.001
Sedentary time	466.56 ± 4.59	476.04 ± 5.63	461.59 ± 5.45	p = 0.003
race (%)				p < 0.001
Mexican American	14.35%	17.53%	12.29%	
Non-Hispanic Black	13.92%	15.69%	12.77%	
Non-Hispanic White	56.30%	51.51%	59.40%	
Other Hispanic	7.25%	7.58%	7.03%	
Other Race	8.18%	7.69%	8.50%	
gender (%)				p = 0.685
male	52.03%	51.54%	52.34%	
female	47.97%	48.46%	47.66%	
activity (%)				p = 0.023
active	79.54%	77.76%	80.70%	
inactive	20.46%	22.24%	19.30%	
Hyperlipemia (%)				p < 0.001
hyperlipemia	8.75%	14.89%	4.76%	
Non-hyperlipemia	91.25%	85.11%	95.24%	

Mean ± SE for continuous variables. SII, systemic immune-inflammation index; *WBC* white blood cell, *PIR* family poverty ratio, *BMI* body mass index, *NHANES* national health and nutrition examination survey. p < 0.05 was considered statistically significant.

### Association between log SII and obesity

The data presented in Table 2 exemplify a robust relationship between log SII and obesity. This association remained statistically significant across all models: unadjusted model 1(OR: 1.890, p < 0.001), minimally adjusted model 2 (OR: 1.993, p < 0.001), and fully adjusted model 3 (OR: 1.280, p < 0.001). To enhance sensitivity, we stratified the log SII values into quartiles. Elevated ORs and significant p-values between SII and obesity were observed in higher quartiles compared to the lowest quartile. Similarly, the trend test showed a significant p-value in all three models (p < 0.001).

**Table 2:** The association between log SII and obesity.

	Model 1	Model 2	Model 3
	OR (95%CI) p Value	OR (95%CI) p Value	OR (95%CI) p Value
Log SII	1.890(1.698–2.104) p < 0.001	1.993(1.788–2.221) p < 0.001	1.280(1.123–1.459) p < 0.001
SII quartiles			
Quartile 1	Reference	Reference	Reference
Quartile 2	1.431(1.224–1.674) p < 0.001	1.503(1.282–1.763) p < 0.001	1.275(1.080–1.506) p = 0.004
Quartile 3	2.029(1.738–2.368) p < 0.001	2.179(1.857–2.556) p < 0.001	1.604(1.352–1.902) p < 0.001
Quartile 4	2.549(2.179–2.968) p < 0.001	2.776(2.362–3.262) p < 0.001	1.561(1.291–1.887) p < 0.001
p For Trend	p < 0.001	p < 0.001	p < 0.001

Model 1, without adjusting for any covariates; Model 2, adjusted for age, gender, and race; Model 3, adjusted for age, gender, race, PIR, activity level, white blood cell count, hyperlipidemia, and sedentary time. Multivariate logistic regression analysis was used to calculate odds ratio values and 95% confidence intervals.95% CI, 95% confidence interval; *OR*, odds ratio, *SII* systemic immunity-inflammation index. p < 0.05 was considered statistically significant.

### Linear regression analysis

To assess the relationship between obesity and Log SII, we established three linear regression models for sensitivity analysis (Table 3). The results of all models showed a significant association between obesity and Log SII (P < 0.001), further confirming the impact of obesity on immune inflammation. By calculating the intercepts and regression coefficients, we found that the average values and standard errors of log SII in the non-obese group (5.922 ± 0.009, 5.730 ± 0.049, 4.709 ± 0.058) were lower than those in the obese group (6.095 ± 0.014, 5.907 ± 0.014, 4.755 ± 0.012).

**Table 3:** Linear Regression Models for sensitivity analysis.

Log SII	Model 1	Model 2	Model 3
Obesity	5.922 ± 0.009	5.730 ± 0.049	4.709 ± 0.058
Non-obesity	6.095 ± 0.014	5.907 ± 0.014	4.755 ± 0.012
p value	p < 0.001	p < 0.001	p < 0.001

Mean ± SE for Log SII. Model 1, without adjusting for any covariates; Model 2, adjusted for age, gender, and race; Model 3, adjusted for age, gender, race, PIR, activity level, white blood cell count, hyperlipidemia, and sedentary time. Multivariate linear regression analysis was used to calculate regression coefficients and standard errors.

### Subgroup analysis

Subgroup analysis demonstrated variable associations between log SII and obesity across different categories. Specifically, the results proved significant in females (OR: 1.419, 95% CI: 1.171–1.719, adjusted p = 0.002) but not males. Additionally, significant differences emerged in the standard white blood count group (OR: 1.831, 95% CI: 1.616–2.075, adjusted p = 0.002) and in the PIR > 1 group (OR: 1.359, 95% CI: 1.155–1.597, adjusted p = 0.002). Conversely, no relationship was found for individuals with a high or low white blood cell count or in the PIR ≤ 1 group. Remarkable associations were also identified in non-Hispanic whites (OR: 1.957; 95% CI: 1.281–2.990; adjusted p = 0.008), Mexican Americans (OR: 1.494; 95% CI: 1.143–1.951; adjusted p = 0.011), the active group (OR: 1.238; 95% CI: 1.067–1.436; adjusted p = 0.014), the inactive group (OR: 1.435; 95% CI: 1.084–1.901; adjusted p = 0.027), and the non-hyperlipidemia group (OR: 1.275; 95% CI: 1.113–1.460; adjusted p = 0.002). Interaction analysis indicated significant interactions between white blood cell count (P < 0.001) and log SII relative to adiposity. However, gender, PIR, age, race, activity level, hyperlipidemia, and sedentary time did not interact significantly. (Table 4).

**Table 4:** Subgroup analysis for the association between log SII and obesity.

Log SII	Model3			
	OR (95%CI)	P value	P for BH	P for interaction
Genger				p = 0.186
male	1.166(0.973–1.398)	p = 0.096	p = 0.157	
female	1.419(1.171–1.719)	p < 0.001	p = 0.002	
Rage				p = 0.543
Non-Hispanic Black	1.136(0.862–1.497)	p = 0.366	p = 0.421	
Non-Hispanic White	1.957(1.281–2.990)	p = 0.002	p = 0.008	
Mexican American	1.494(1.143–1.951)	p = 0.003	p = 0.011	
Other Race	1.362(0.901–2.058)	p = 0.143	p = 0.204	
Other Hispanic	1.106(0.878–1.394)	p = 0. 393	p = 0.430	
Activity				p = 0.248
active	1.238(1.067–1.436)	p = 0.005	p = 0.014	
inactive	1.435(1.084–1.901)	p = 0.012	p = 0.027	
Hyperlipemia				p = 0.134
hyperlipemia	1.608(0.956–2.704)	p = 0.074	p = 0.142	
Non-hyperlipemia	1.275(1.113–1.460)	p = 0.001	p = 0.002	
WBC				p < 0.001
< 4	1.515(0.706–3.250)	p = 0.287	p = 0.348	
4–10	1.831(1.616–2.075)	p < 0.001	p = 0.002	
> 10	1.076(0.671–1.726)	p = 0.761	p = 0.761	
Sedentary time				p = 0.896
Q1	1.393(1.096–1.771)	p = 0.007	p = 0.017	
Q2	1.237(0.964–1.585)	p = 0.094	p = 0.157	
Q3	1.213(0.989–1.489)	p = 0.064	p = 0.134	
PIR				p = 0.235
≤ 1	1.162(0.928–1.454)	p = 0.191	p = 0.244	
> 1	1.359(1.155–1.597)	p < 0.001	p = 0.002	

Model 3 (adjusted for age, gender, race, PIR, activity, WBC, Hyperlipemia, and Sedentary time). Multivariate logistic regression was employed to calculate beta values and 95% confidence intervals. 95% CI, 95% confidence interval; *OR* odds ratio, *SII* systemic immunity-inflammation index, *WBC* white blood cell, *PIR* family poverty ratio; P for BH, the p-values corrected using the Benjamini–Hochberg method; p < 0.05 was considered statistically significant.

 In addition, we also plotted smoothed curve fits of Log SII in each subgroup of the population, thus highlighting and visualizing the different effects of SII on different populations. The entire figure can be found as Supplementary Figure S1-S19 online.

### Threshold effect analysis and smooth curve

**Fig. 2:**
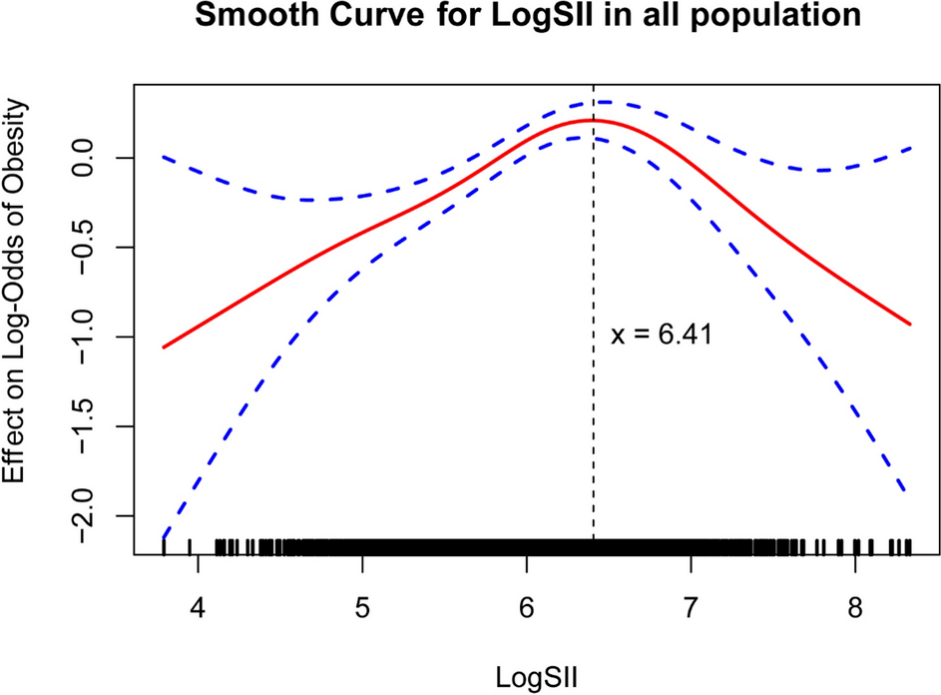
The relationship between obesity and log SII is non-linear, smooth curve fit is shown by the solid red line, with the 95% confidence interval represented by the blue band.

A generalized additive model was constructed, and a smoothed fitting curve was plotted (Fig. 2), revealing an inverted U-shaped relationship.

The results of the threshold effect analysis revealed a critical turning point at log SII = 6.410. Below this threshold, the relationship between log SII and obesity remained statistically significant and positive (OR: 1.702; 95% CI: 1.445–2.093; p < 0.001). However, above this threshold, the association became negative (OR: 0.502; 95% CI: 0.358–0.705; p < 0.001). The likelihood ratio test of the two-stage generalized additive model also indicated significance (p < 0.001), suggesting that the model better captured this association between log SII and obesity (Table 5).

**Table 5:** Threshold effect analysis results of the relationship between log SII and obesity.

	Log SII	OR (95%CI)	p value
Model 1		1.890(1.698–2.006)	p < 0.001
Model 3		1.280(1.123–1.459)	p < 0.001
LR test			p < 0.001
Model 4			
Inflection point		6.410	
< 6.410		1.702(1.445–2.093)	p < 0.001
> 6.410		0.502(0.358–0.705)	p < 0.001
LR test			p < 0.001

SII, systemic immunity-inflammation index; 95% CI, 95% confidence interval; OR, odds ratio; Model 1, without adjusting for any covariates; Model 3, adjusted for age, gender, race, PIR, activity, WBC, Hyperlipemia, and Sedentary time; Model 4, segmented Logistic Regression Models Adjusted for Age, Sex, Race, PIR, Activity, Leukocytes, Hyperlipidemia, and Sedentary Time; LR test, log-likelihood ratio test; p < 0.05 was considered statistically significant.

### Mediation analysis of the SII-obesity relationship

**Fig. 3:**
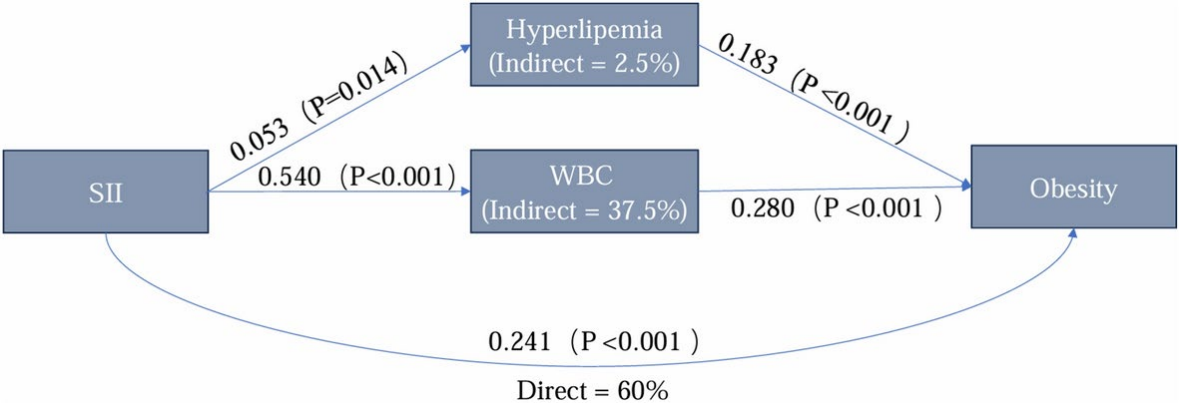
Mediational Pathway of SII on Obesity through Hyperlipemia and WBC.

Through mediation analysis, we identified hyperlipidemia and WBC as significant mediators in the relationship between SII and adolescent obesity. The specific path diagram is shown in Fig. 3The total effect of SII on obesity was 0.402, with a direct effect of 0.241, accounting for 60.0% of the total effect. The indirect effects mediated by WBC and hyperlipidemia were 0.151 (37.5% of the total effect, P < 0.001) and 0.010 (2.5% of the total effect, P = 0.046), respectively. These findings highlight the pivotal role of inflammation and immune responses, particularly WBC elevation, in mediating the effects of SII on obesity. Pathway analysis further revealed that SII had a stronger direct effect on WBC (a2 = 0.540, P < 0.001) compared to hyperlipidemia (a1 = 0.053, P = 0.014), while WBC (b2 = 0.280, P < 0.001) had a more pronounced influence on obesity than hyperlipidemia (b1 = 0.183, P < 0.001). These results suggest that WBC plays a dominant role in the SII-obesity relationship, while hyperlipidemia contributes more modestly. These results indicate that white blood cells play a dominant role in mediating the relationship between SII and obesity, while hyperlipidemia has a relatively smaller impact on obesity. Other covariates in the model showed significant direct effects on obesity but did not exhibit substantial mediating effects.

## Discussion

In recent years, numerous studies have suggested that the interaction between obesity and chronic inflammation involves complex mechanisms such as immune responses and metabolic changes. Adipose tissue is not only a site for energy storage but also acts as an immune-active organ, secreting various pro-inflammatory mediators collectively known as adipokines^33^ (e.g., adiponectin, leptin, TNF-α, IL-1β, and IL-6). Dysregulation of these adipokines results in an inflammatory environment within adipose tissue. As adipocytes expand and fat accumulates, the infiltration of immune cells, especially macrophages, further exacerbates local and systemic inflammation^34^. At the same time, the combined effects of adipokines and pro-inflammatory cytokines released by macrophages may impair insulin signaling pathways^35^, driving the onset of obesity and its complications, creating a vicious cycle.

In addition to macrophages, neutrophils, platelets, and lymphocytes also play significant roles in the chronic inflammation triggered by obesity^36,37^. Neutrophils can also exacerbate local and systemic inflammation by releasing pro-inflammatory factors such as TNF-α and IL-1β^14^. Platelets promote the recruitment of immune cells by secreting cytokines and chemokines. The functions of T cells and B cells also change with metabolic alterations. The metabolic disorders induced by obesity lead to changes in T cell subsets, particularly an increase in pro-inflammatory Th17 cells, while regulatory T cells (Treg) decrease, thereby weakening immune tolerance and exacerbating inflammation^38^. Meanwhile, B cells in obese individuals typically exhibit hyperactivation, producing more pro-inflammatory cytokines, further promoting immune dysregulation^16^. Overall, obesity leads to changes in immune cell function, making these cells more active and less regulated, increasing immune cell infiltration in organs, ultimately intensifying systemic inflammation and increasing the risk of autoimmune diseases, infections, and cancer.

In this complex network of immune response and metabolic changes, the SII (Systemic Inflammation Index) integrates the changes in neutrophils, platelets, and lymphocytes, quantifying the relationships between these three cell types, providing a more precise assessment of the interaction between obesity and inflammation. Our study established three logistic regression models, progressively controlling for confounding factors to evaluate the relationship between log SII and adolescent obesity. The results showed a significant positive correlation between log SII and adolescent obesity, even in the fully adjusted model (model 3) (P < 0.001, OR: 1.280 (1.123–1.459)). Subsequent analysis using quartiles of SII revealed that, compared to the Q1 range, adolescents in the Q2-Q4 ranges had a higher risk of obesity, with significant trend tests in all three models (p < 0.001). These experimental results confirm that SII can effectively assess the relationship between obesity and chronic inflammation.

However, smooth curve fitting and threshold effect analysis showed that the relationship between log SII and obesity is non-linear, exhibiting an inverted U-shaped curve with a turning point at 6.410. Before SII reaches 6.410, log SII is positively correlated with obesity risk, with an OR value of 1.702(1.445–2.093), suggesting that as SII increases and systemic inflammation intensifies, adipokines and pro-inflammatory cytokines released by macrophages may impair insulin signaling pathways and induce insulin resistance, driving the onset of obesity and its complications^16^. After exceeding the threshold, log SII shows a negative correlation with obesity risk, with an OR value of 0.502(0.358–0.705). This inverse relationship may be related to several mechanisms. On one hand, high levels of SII reflect severe systemic inflammation, which is often accompanied by metabolic disorders, manifested as increased energy expenditure, abnormal fat breakdown, and weight loss^39,40^. On the other hand, elevated peripheral pro-inflammatory cytokines act on immune cells around the vagus nerve, which secrete IL-1. Through paracrine effects, IL-1 activates IL-1 receptors on the vagus nerve, ultimately transmitting signals via the vagus nerve, disrupting the normal function of the hypothalamic neuropeptide circuits, leading to reduced appetite and involuntary weight loss^41,42^. Additionally, very high levels of SII may reflect an acute inflammatory state, which, through mechanisms like increased energy expenditure and altered appetite regulation^43,44^, further leads to weight loss and reduces the risk of obesity. Although these mechanisms are not fully understood, they may interact to explain the inverse relationship.

Our study adds a new perspective to the existing literature on the relationship between inflammation and obesity by evaluating the complex relationship between log SII and obesity. Understanding the bidirectional relationship between inflammation and obesity will help develop more personalized approaches to obesity management. Although some studies have explored the relationship between SII and obesity in the adult population in the United States^45,46^, significant differences exist between adults and adolescents in terms of physiological characteristics and obesity definitions. This study fills this gap in adolescent populations, expanding the current research in this field and providing more targeted insights for adolescent obesity.

Additionally, subgroup analysis results showed that the relationship between log SII and obesity differs based on sex, white blood cell count, and family income-to-poverty ratio. We found a strong positive correlation between log SII and obesity in females, but not in males. This may be due to differences in inflammatory oxidized lipid levels between males and females^47^. Premenopausal women have higher plasma levels of oxidized lipids such as 14-HDHA and 12-HEPE, synthesized by 12-lipoxygenase, compared to older women and men. Oxidized lipids, especially 14-HDHA and 12-HEPE synthesized by 12-lipoxygenase, have been shown to suppress inflammation, improve insulin sensitivity, and promote fat metabolism. Higher levels of oxidized lipids may have anti-inflammatory effects by regulating immune cell activity, reducing the release of pro-inflammatory cytokines, and alleviating systemic low-grade inflammation. In addition, studies have shown that ovariectomy in mice increases lung inflammation and decreases oxidized lipid levels^48^, suggesting that sex hormones may mediate the differences we observed^49^. Furthermore, differences in fat distribution and adipose tissue metabolic functions between males and females may also contribute to these observed gender differences^50,51^. Under the combined effects of these mechanisms, females may experience higher levels of inflammation and metabolic disturbances at the same SII value. Therefore, women have a relatively higher risk of obesity when SII is elevated, and the relationship between SII and obesity is more pronounced in females.

In addition, among populations with normal white blood cell counts, log SII is significantly associated with obesity. However, in subjects with higher or lower white blood cell counts, the correlation is not as clear. Our study also found a significant interaction effect between white blood cell count and log SII (P < 0.001), indicating that white blood cell count may mediate the relationship between SII and adolescent obesity. For individuals with normal white blood cell counts, SII can intuitively reflect the connection between chronic low-grade inflammation and obesity. In contrast, when white blood cell counts are abnormal, such as in cases of elevated white blood cell counts, this often accompanies acute inflammatory responses and overactivation of the immune system. When white blood cell counts are too low, a condition known as leukopenia, it may indicate an abnormal state, such as immune suppression due to chemotherapy or autoimmune diseases, abnormal hematopoiesis, viral infections, or malnutrition. Therefore, both high and low white blood cell counts can interfere with the relationship between SII and obesity by affecting immune response intensity and metabolic regulation.

The significant relationship between log SII and obesity also varies between families with different income levels. Some studies suggest that individuals with higher socioeconomic status typically have better access to medical resources and health education, allowing them to more effectively control weight and inflammation caused by other factors^52^. In contrast, lower-income groups may face greater challenges in accessing medical resources and health education, which complicates the relationship between socioeconomic status and obesity and influences the relationship between log SII and adolescent obesity.

Significant positive correlations were also observed in non-Hispanic White, Mexican American, active, inactive, and non-hyperlipidemia groups. These results highlight the importance of developing personalized interventions based on individual characteristics. For example, increasing physical activity, improving dietary habits, and strengthening health education could reduce inflammation and control weight in high-risk subgroups^26^.

In subsequent research, we explored the underlying mechanisms of the association between SII and adolescent obesity using mediation analysis, identifying hyperlipidemia and WBC as significant mediators. Among these, WBC accounted for 37.5% of the total effect of SII on obesity, while hyperlipidemia accounted for 2.5%. These findings suggest that inflammation and immune responses, particularly the elevation of WBC, play a critical role in the relationship between SII and obesity. This aligns with existing evidence that systemic inflammation, quantified by SII, drives obesity by altering immune cell function and promoting insulin resistance. However, the differing contributions of direct and mediated effects indicate that SII may influence obesity through multiple pathways beyond the mediators examined in this study. This highlights the need for future research to incorporate other potential mediators, such as cytokine levels or metabolic markers, to comprehensively evaluate the complex interactions between inflammation and obesity.

Our study has several limitations that should be considered. First, due to the cross-sectional design, we cannot establish any causal relationships. Second, some variables in NHANES rely on self-report, which may introduce recall or reporting bias, limiting the accuracy of the results. Obesity is a multifactorial disease. Although our analysis adjusted for various covariates, some unmeasured confounders (such as genetic background, early life environment, and psychological stress) were not considered. These confounders may affect the interpretation of the results. Thus, our study may not fully describe the relationship between SII and obesity. Future research should account for these unmeasured confounders and conduct longitudinal studies to verify our findings.

## Conclusion

Based on the National Health and Nutrition Examination Survey (NHANES) data, this study found a complex U-shaped relationship between the Systemic Immune-Inflammation Index (SII) and adolescent obesity, suggesting that SII plays a key role in the interaction between inflammation and obesity. This finding provides a new index for quantifying systemic inflammation in obesity, advancing our evaluate of adolescent obesity risk. Future research should focus on validating the causal relationship between SII and obesity, exploring the mechanisms by which SII affects obesity through immune cells, and evaluating interventions targeting SII levels (such as anti-inflammatory treatments or lifestyle changes) for obesity prevention. These efforts will help develop more personalized approaches to obesity prevention and treatment.

## Supplementary Information

Supplementary Information.

## Data Availability

The data supporting the findings of this study are available from the corresponding author upon request. Publicly available datasets from the National Health and Nutrition Examination Survey (NHANES) can be accessed through online repositories: https://www.cdc.gov/nchs/nhanes/.

## Abbreviations

NHANES: National health and nutrition examination survey
CI: Confidence interval
OR: Odds ratios
SII: Systemic Immune-Inflammation Index
BMI: Body mass index
PIR: Poverty income ratio
SE: Standard error
ANOVA: Analysis of variance
GAM: Generalized additive model
