# Supplementary material for: Association between systemic immune inflammation index and adolescent obesity in a cross-sectional analysis
Source: Sci Rep. 2025 Feb 22;15:6439. doi: 10.1038/s41598-025-91125-6 (PMC11846860; doi:10.1038/s41598-025-91125-6)
Supplement: Supplementary file 1 — Supplementary Information. [file 41598_2025_91125_MOESM1_ESM.pdf]

Supplementary figure S1.Smooth Curve for LogSII in Female group

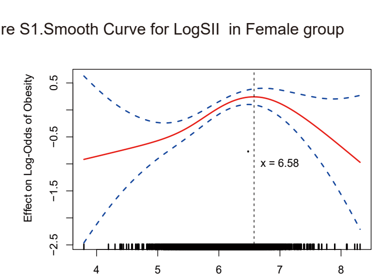

Supplementary figure S1 represent the relationship between Log SII and obesity risk in female group. Model was generalized additive models with covariates including all covariates except the basis of grouping. Log-Odds, logarithmic odds; smooth curve fit is shown by the solid red line, with the 95% confidence interval represented by the blue.

Supplementary figure S2.Smooth Curve for LogSII in Male group

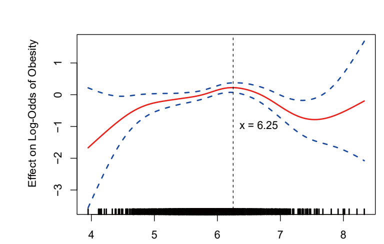

Supplementary figure S2 represent the relationship between Log SII and obesity risk in male group. Model was generalized additive models with covariates including all covariates except the basis of grouping. Log-Odds, logarithmic odds; smooth curve fit is shown by the solid red line, with the 95% confidence interval represented by the blue.

Supplementary figure S3.Smooth Curve for LogSII in Active group

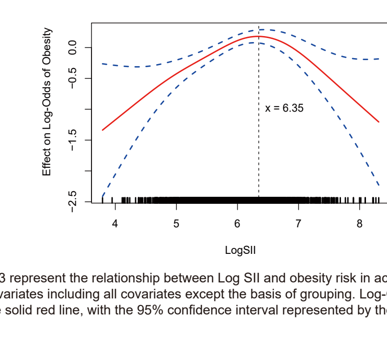

Supplementary figure S3 represent the relationship between Log SII and obesity risk in active group. Model was generalized additive models with covariates including all covariates except the basis of grouping. Log-Odds, logarithmic odds; smooth curve fit is shown by the solid red line, with the 95% confidence interval represented by the blue.

Supplementary figure S4.Smooth Curve for LogSII in Inactive group

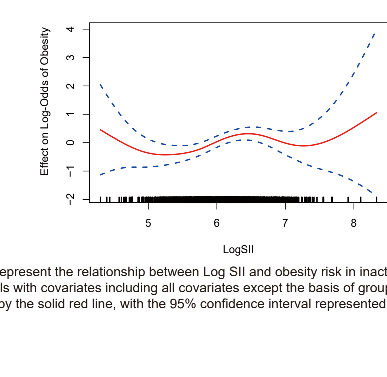

Supplementary figure S4 represent the relationship between Log SII and obesity risk in inactive group. Model was generalized additive models with covariates including all covariates except the basis of grouping. Log-Odds, logarithmic odds; smooth curve fit is shown by the solid red line, with the 95% confidence interval represented by the blue.

Supplementary figure S5.Smooth Curve for LogSII in Mexican American

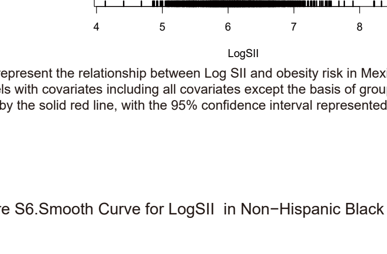

Supplementary figure S5 represent the relationship between Log SII and obesity risk in Mexican American. Model was generalized additive models with covariates including all covariates except the basis of grouping. Log-Odds, logarithmic odds; smooth curve fit is shown by the solid red line, with the 95% confidence interval represented by the blue.

Supplementary figure S6.Smooth Curve for LogSII in Non−Hispanic Black

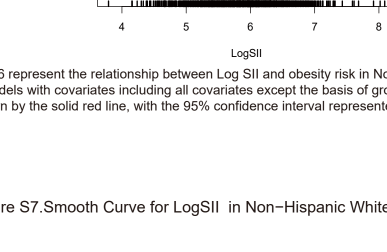

Supplementary figure S6 represent the relationship between Log SII and obesity risk in Non−Hispanic Black. Model was generalized additive models with covariates including all covariates except the basis of grouping. Log-Odds, logarithmic odds; smooth curve fit is shown by the solid red line, with the 95% confidence interval represented by the blue.

Supplementary figure S7.Smooth Curve for LogSII in Non−Hispanic White

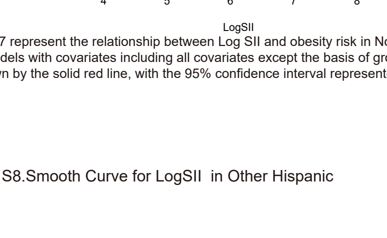

Supplementary figure S7 represent the relationship between Log SII and obesity risk in Non−Hispanic White. Model was generalized additive models with covariates including all covariates except the basis of grouping. Log-Odds, logarithmic odds; smooth curve fit is shown by the solid red line, with the 95% confidence interval represented by the blue.

Supplementary figure S8.Smooth Curve for LogSII in Other Hispanic

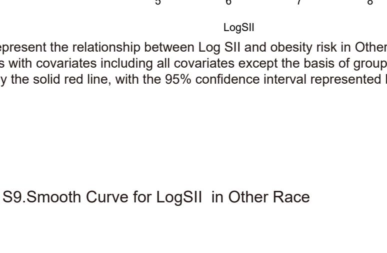

Supplementary figure S8 represent the relationship between Log SII and obesity risk in Other Hispanic. Model was generalized additive models with covariates including all covariates except the basis of grouping. Log-Odds, logarithmic odds; smooth curve fit is shown by the solid red line, with the 95% confidence interval represented by the blue.

Supplementary figure S9.Smooth Curve for LogSII in Other Race

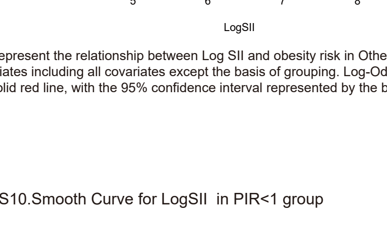

Supplementary figure S9 represent the relationship between Log SII and obesity risk in Other Race. Model was generalized additive models with covariates including all covariates except the basis of grouping. Log-Odds, logarithmic odds; smooth curve fit is shown by the solid red line, with the 95% confidence interval represented by the blue.

Supplementary figure S10.Smooth Curve for LogSII in PIR<1 group

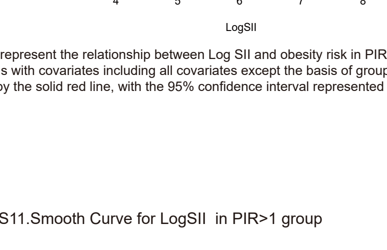

Supplementary figure S10 represent the relationship between Log SII and obesity risk in PIR<1 group. Model was generalized additive models with covariates including all covariates except the basis of grouping. Log-Odds, logarithmic odds; smooth curve fit is shown by the solid red line, with the 95% confidence interval represented by the blue.

Supplementary figure S11.Smooth Curve for LogSII in PIR>1 group

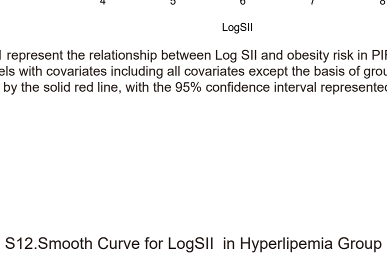

Supplementary figure S11 represent the relationship between Log SII and obesity risk in PIR>1 group. Model was generalized additive models with covariates including all covariates except the basis of grouping. Log-Odds, logarithmic odds; smooth curve fit is shown by the solid red line, with the 95% confidence interval represented by the blue.

Supplementary figure S12.Smooth Curve for LogSII in Hyperlipemia Group

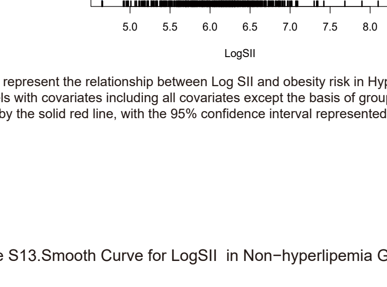

Supplementary figure S12 represent the relationship between Log SII and obesity risk in Hyperlipemia Group. Model was generalized additive models with covariates including all covariates except the basis of grouping. Log-Odds, logarithmic odds; smooth curve fit is shown by the solid red line, with the 95% confidence interval represented by the blue.

Supplementary figure S13.Smooth Curve for LogSII in Non−hyperlipemia Group

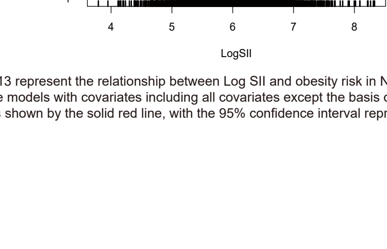

Supplementary figure S13 represent the relationship between Log SII and obesity risk in Non−hyperlipemia Group. Model was generalized additive models with covariates including all covariates except the basis of grouping. Log-Odds, logarithmic odds; smooth curve fit is shown by the solid red line, with the 95% confidence interval represented by the blue.

Supplementary figure S14.Smooth Curve for LogSII in Sedentary Q1 group

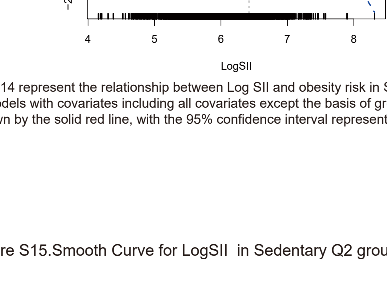

Supplementary figure S14 represent the relationship between Log SII and obesity risk in Sedentary Q1 group. Model was generalized additive models with covariates including all covariates except the basis of grouping. Log-Odds, logarithmic odds; smooth curve fit is shown by the solid red line, with the 95% confidence interval represented by the blue.

Supplementary figure S15.Smooth Curve for LogSII in Sedentary Q2 group

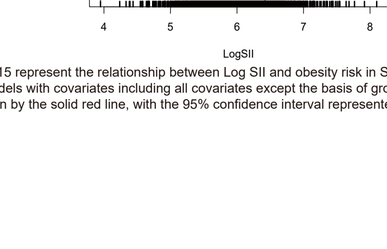

Supplementary figure S15 represent the relationship between Log SII and obesity risk in Sedentary Q2 group. Model was generalized additive models with covariates including all covariates except the basis of grouping. Log-Odds, logarithmic odds; smooth curve fit is shown by the solid red line, with the 95% confidence interval represented by the blue.

Supplementary figure S16.Smooth Curve for LogSII in Sedentary Q3 group

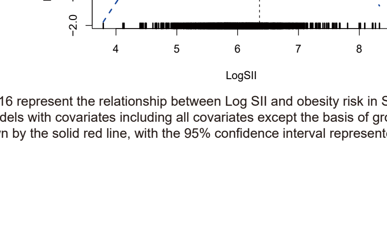

Supplementary figure S16 represent the relationship between Log SII and obesity risk in Sedentary Q3 group. Model was generalized additive models with covariates including all covariates except the basis of grouping. Log-Odds, logarithmic odds; smooth curve fit is shown by the solid red line, with the 95% confidence interval represented by the blue.

Supplementary figure S17.Smooth Curve for LogSII in WBC<4 group

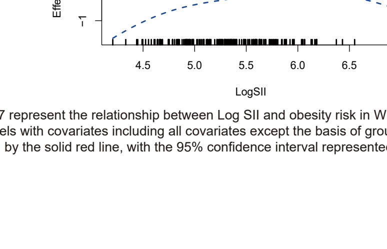

Supplementary figure S17 represent the relationship between Log SII and obesity risk in WBC<4 group. Model was generalized additive models with covariates including all covariates except the basis of grouping. Log-Odds, logarithmic odds; smooth curve fit is shown by the solid red line, with the 95% confidence interval represented by the blue.

Supplementary figure S18.Smooth Curve for LogSII in 4<WBC<10 group

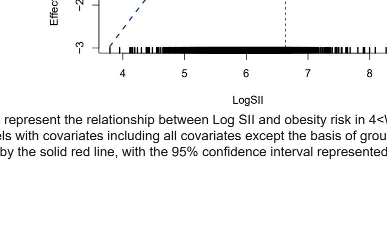

Supplementary figure S18 represent the relationship between Log SII and obesity risk in 4<WBC<10 group. Model was generalized additive models with covariates including all covariates except the basis of grouping. Log-Odds, logarithmic odds; smooth curve fit is shown by the solid red line, with the 95% confidence interval represented by the blue.

Supplementary figure S19.Smooth Curve for LogSII in 10<WBC group

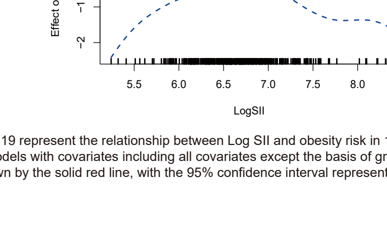

Supplementary figure S19 represent the relationship between Log SII and obesity risk in 10<WBC group. Model was generalized additive models with covariates including all covariates except the basis of grouping. Log-Odds, logarithmic odds; smooth curve fit is shown by the solid red line, with the 95% confidence interval represented by the blue.
